# The Potential of Hemp Extracts to Modify the Course of Oxidative-Stress Related Conditions

**DOI:** 10.3390/plants13121630

**Published:** 2024-06-13

**Authors:** Katarina Bijelić, Branislava Srdjenović Čonić, Blagoje Prpa, Vladimir Pilija, Saša Vukmirović, Nebojša Kladar

**Affiliations:** 1 Department of Pharmacy, Faculty of Medicine, University of Novi Sad, Hajduk Veljkova 3, 21000 Novi Sad, Serbia; katarina.bijelic@mf.uns.ac.rs (K.B.); blagoje.prpa@mf.uns.ac.rs (B.P.); nebojsa.kladar@mf.uns.ac.rs (N.K.); 2 Faculty of Medicine, Center for Medical and Pharmaceutical Investigations and Quality Control, University of Novi Sad, Hajduk Veljkova 3, 21000 Novi Sad, Serbia; 3 Institute of Forensic Medicine, Clinical Center Vojvodina, Faculty of Medicine, University of Novi Sad, Hajduk Veljkova 3, 21000 Novi Sad, Serbia; vladimir.pilija@mf.uns.ac.rs; 4 Faculty of Medicine, Department of Pharmacology, Toxicology and Clinical Pharmacology, University of Novi Sad, Hajduk Veljkova 3, 21000 Novi Sad, Serbia; sasa.vukmirovic@mf.uns.ac.rs

**Keywords:** hemp, hemp teas, polyphenolics, antioxidative, antihyperglycemic

## Abstract

The leaves of industrial hemp, which have long been considered as a waste product, have been proven to contain numerous compounds that possess potential biological activity. One of the most interesting groups of compounds present are polyphenolic compounds, which, due to their specific structure, have a pronounced antioxidant and antihyperglycemic potential. This study aimed to detect biological activity, including antioxidant and antihyperglycemic potential, of water and water–alcoholic extracts of five commercially available hemp teas, followed by phytochemical profiling. Hemp aqueous and ethanolic extracts demonstrate potent antioxidant properties. Ethanol extracts are better scavengers of DPPH• and OH•, while aqueous extracts neutralize NO• better. Both types of extracts exhibit antioxidant potential in the catalase test and moderate XOD inhibition. Furthermore, aqueous extracts are potent α-amylase inhibitors, while ethanolic extracts demonstrate stronger anti-α-glucosidase activity, suggesting therapeutic potential for chronic diseases like insulin resistance or diabetes. Further detailed chemical characterization and in vivo studies are needed to validate these findings.

## 1. Introduction

Hemp (*Cannabis sativa* L., Cannabaceae), a plant with a cultivation history of more than 5000 years, represents a multipurpose crop. So far, this species has been utilized across a spectrum of sectors, such as agriculture, cosmetics, paper manufacturing and biofuel production [1,2]. Industrial hemp (non-psychoactive type), characterized by its minimal levels of psychoactive Δ^9^-tetrahydrocannabinol (Δ^9^-THC), was predominantly grown for the fabrication of high-quality fiber materials. However, due to the presence of more than 500 biologically active compounds which show beneficial effect on human health, it has also found application in medicine and the food industry [3,4]. The most characteristic compounds present in hemp are phytocannabinoids that belong to the group of terpeno-phenolic compounds. To date, more than 100 phytocannabinoids have been successfully chemically characterized. These compounds, through their interaction with the endocannabinoid system, serve as a cornerstone for the therapeutic use of *Cannabis* species [5,6].

Apart from cannabinoids, another highly abundant and significant group of compounds are polyphenols, especially flavonoids such as flavanones, flavanols and isoflavones [1,7]. Polyphenolic compounds, due to their characteristic structure and the presence of hydroxyl groups, act as powerful antioxidants and reduce the levels of reactive oxygen species and therefore have a significant role in the prevention of numerous diseases and conditions such as insulin resistance, diabetes mellitus II, cardiovascular diseases and hypertension and numerous neurodegenerative diseases. Plants abundant in polyphenols, like hemp, are frequently utilized in food and dietary supplements due to their wide spectra of beneficial effects [8,9]. Taking into account the increasing prevalence of metabolic diseases linked to antioxidant stress, products obtained from hemp (such as extracts) could serve as a significant resource for their prevention. Although CBD and polyphenols act synergistically and contribute to an increase in antioxidant capacity, recent European Food Safety Agency (EFSA) reports suggest numerous negative effects of CBD consumption on human health. Therefore, from a consumer safety perspective, it would be more rational to use hemp extracts obtained by application of polar solvents, which are less favorable to liposoluble cannabinoids such as CBD [10,11,12].

When it comes to hemp products, according to some research, fibers, oilseed and pharmaceuticals have the greatest marketing potential [13]. However, hemp-based teas can often be found on the market today. The leaves (teas) are generally considered a waste product during fiber production, but the abundance of polyphenolic compounds makes them interesting for investigating potential biological effects and increasing the utilization of hemp [12,14]. Therefore, the aims of this research were the chemical characterization of aqueous and ethanol hemp extracts in terms of phenolic and flavonoid compounds as well as the examination of their in vitro biological activity in terms of antioxidant potential and antihyperglycemic potential.

## 2. Results

### 2.1. Chemical Characterization of Hemp Extracts

The application of ethanol (70%, *v*/*v*) for extraction resulted in extract yield ranging from 9.44 to 19.99%, whereas in case of water extraction the extraction yield was slightly higher and ranged from 12.7 to 29.56% (Table 1).

The previously conducted studies have identified polyphenolic compounds as an important class of antioxidants of natural origin. The content of total phenolics (Table 1) in the water–ethanolic extracts ranged from 34 to 63 mg of gallic acid equivalents per gram of dry extract (mg GAE/g d.e.), whereas in the aqueous extracts it ranged from 24 to 72 mg GAE/g d.e. However, there were no statistically significant differences in the recorded amount of total phenolics between the two evaluated types of extracts (z score = 1.82, *p* = 0.07). The application of water as an extractant resulted in total flavonoids ranging from 7 to 19 mg of quercetin equivalents per gram of dry extract (mg QE/g d.e.), while the water–alcoholic extracts contained 6–34 mg QE/g d.e. (Table 1). Similarly, as in the case of total phenolics, no statistically significant differences were found in the total amount of flavonoids (z score = 0.57, *p* = 0.57) regarding the solvent applied for the extraction.

Through preliminary chemical characterization, it was determined that in 60% of the tested samples the content of total phenolics was higher in the water–ethanolic extracts, while, on the other hand, the same percentage of examined water extracts contained higher quantities of total flavonoids. The recorded variability could be explained by the great diversity of the mentioned classes of secondary metabolites in terms of chemical structure, which further affects solubility in different solvents. Also, in the case of flavonoids, the possibility of the occurrence of certain compounds in the form of aglycones and corresponding glycosides strongly emphasizes the influence of the choice of extractant on the chemical profile of the obtained extract. The herbal material used in our research were commercially available products (hemp teas) intended for preparation by consumers in the form in which they were sold. Specifically, no grinding was advised to consumers prior to beverage preparation, and, in order to provide a realistic insight into the quantities of secondary metabolites which could be extracted from such material, we have also not applied a grinding procedure to the teas prior to extraction.

However, the visual inspection of the purchased products indicated evident differences in the degree of grinding of the herbal material, as well as no uniformity regarding the presence of different plant parts (leaves, inflorescences, stems). The aforementioned factors are well known to affect the amounts of secondary metabolites being extracted. In the research carried out by Tiago et al., 2022 [4], the highest content of flavonoids was extracted using non-polar solvents compared to more polar extractants, which is in accordance with our study indicating the highest concentration of total flavonoids in the water–ethanolic extract; however, in 60% of cases, the flavonoid content was higher in the aqueous extracts. This confirms the previously mentioned variable solubility of flavonoids due to their varied structure. Furthermore, the intensity of biosynthesis of phenolics and flavonoids (as well as other secondary metabolites) is highly influenced by the abiotic environmental factors characteristic of the plant’s habitat and should be taken into account when chemically profiling the same species of different geographical origins or comparing the obtained results with the results of previously conducted studies [15]. In the research carried out by Essien 2011 [16], the total phenolic content of the hemp leaves was significantly lower (0.09–0.56 mg GAE/g d.e) than the results obtained in our research. One of the reasons for the aforementioned was the use of solvents of different polarity (methanol, acetone and their 50% aqueous solutions), while, on the other hand, in the current study, a longer period of extraction (in the case of water–ethanolic extraction, a 24 h maceration was applied), as well as higher temperature (in the case of water extraction), could have been the reason for the higher obtained content of phenolic compounds. Another study reported a total phenolic content in the inflorescences of industrial hemp ranging from 10.51 to 52.58 mg GAE/g [7]. These results were similar to those obtained in our study, which indicates a higher percentage of the presence of inflorescences in the evaluated tea samples. The research conducted by Drinić et al., 2018 [17] demonstrated that a water–ethanolic solution (50%, *v*/*v*) extracted the highest amount of phenolic compounds from the aerial part of hemp, compared to 90%, 70%, 30% ethanol and pure water. Phenolic compounds exhibit greater polarity in relation to cannabinoids and terpenes due to their higher oxygen–carbon ratio.

Variability in polarity renders the phenolic fraction less soluble in pure ethanol (non-polar solvents). This implies that utilizing a combination of these solvents would be the most advantageous, as it could concomitantly mitigate ethanol’s toxicity while enhancing the safety of the resulting extracts [18].

The results of detailed chemical characterization regarding the content of polyphenol components are shown in Table 1. Four out of ten analyzed components were below the analytical method detection limit (gallic acid (GA), quercetin (Qe), rosmarinic acid (RA) and rutin (R), while quercitrin (Qt) and ferulic acid (FA) were present in only one of the examined extracts.

Caffeic acid (CA) was present in almost all extracts. However, there was no statistically significant difference in the determined content regarding the type of the obtained extracts (z score = 0.85, *p* = 0.39). Chlorogenic acid (CHA) was quantified in three out of five aqueous extracts and in only one ethanolic. The presence of a significantly higher amount of this acid in the ethanol extracts indicates that chlorogenic acid has a higher affinity for ethanol, which, further, does not explain the absence of this compound in the other ethanol extracts. As the leaves were until recently considered a waste product, very little research was devoted to the identification of polyphenolic compounds. In the paper published by Mpakos et al., 2024 [19], the influence of various factors during the extraction on the isolation of the bioactive components of hemp was investigated. The results of the HPLC-DAD analysis indicated a high abundance of ursolic acid, while chlorogenic acid, rutin, apigenin, luteolin and pelargonin were present but in somewhat lower amounts. Also, in a paper published by Izzo et al., 2020 [7], the polyphenolic profile of extracts obtained from Cannabis inflorescences was examined. The presence of chlorogenic, caffeic, p-coumaric and gallic acids was recorded. Of the mentioned phenolic acids, ferulic acid was the dominant compound, while caffeic acid was significantly less abundant, which was not in accordance with our results. For the extraction of inflorescences in this work, methanol was used, which indicates a different affinity of phenolic compounds for solvents, as well as variable polyphenolic profiles between inflorescences and leaves. Moreover, the analysis of the flavonoids rutin and quercetin indicated their higher content when compared to our samples.

Previous research has shown that chlorogenic acid (which was present in some of currently studied samples) can affect glucose metabolism and shows some therapeutic potential in diabetes. Also, the influence of caffeic acid and its derivatives on insulin stimulation was observed [20,21,22]. Based on the previously published data and the results of detailed chemical characterization of the tested extracts, the antihyperglycemic potential of industrial hemp could be assumed, but the possibility of the presence of other phenolic and flavonoid compounds which were not identified (but could support this biological activity) must not be ignored. Moreover, given the spectrum of conditions stemming from prolonged diabetes characterized by increased levels of oxidative stress, it is imperative not to overlook the well-documented antioxidant capacity of all phenolic and flavonoid compounds presented in extracts [23].

### 2.2. In Vitro Antioxidant Potential

In recent decades, there has been a notable surge in the exploration of plants and their extracts as promising reservoirs of antioxidant compounds, driven by the escalating incidence of neurodegenerative diseases and metabolic syndromes primarily attributed to oxidative stress. Also, due to their strong antioxidant abilities, plant extracts have found application in the food industry as preservatives of natural origin. In addition, the use of extracts rich in polyphenolic compounds (those that are largely responsible for the manifestation of antioxidant potential) is growing in the cosmetic industry, because they have the potential to mitigate symptoms and impede the progression of various skin disorders. Consumers tend to buy cosmetic products that contain plant extracts more and more, due to the fear of allergies and other adverse reactions caused by the presence of synthetic compounds [10,24,25,26].

#### 2.2.1. Radical Scavenging Capacity (RSC)

The antioxidant potential results of the evaluated hemp extracts are presented in Table 2. It can be observed that ethanolic extracts statistically significantly better neutralize 50% (RSC_50_) of DPPH• (z score = 3.07, *p* = 0.002) and OH• radicals (z score = 3.07, *p* = 0.002), while, in the case of NO• neutralization, aqueous extracts were significantly more effective (z score = 3.41, *p* = 0.001). Regarding the DPPH assay, all analyzed extracts neutralized 50% of free radicals in the tested concentration range. The greatest DPPH• radical scavenging potential was recorded for extracts 2E and 4Aq, with RSC_50_ values of 35.02 µg/mL and 41.95 µg/mL, respectively, which is of significance when compared to the antioxidant potential of the positive controls—quercetin dihydrate (RSC_50_ = 1.04 µg/mL) and propyl gallate (RSC_50_ = 0.83 µg/mL) determined under the same experimental conditions. Extract 4Aq contained the highest amount of polyphenolic compounds. Also, the IC_50_ for the DPPH indicated strong antioxidant potential, which confirms the conclusion from the research published by Teixeira et al., 2017 [27] that there is a high correlation between the amount of polyphenolic compounds and antioxidant capacity. Extract 3Aq was the most effective in scavenging NO• radicals (RSC_50_ = 360 µg/mL), which could be considered as moderate antioxidant activity if compared with propyl gallate (RSC_50_ = 12.45 µg/mL). Based on these values, it can be concluded that polyphenolic compounds are not the only ones in these extracts that affect the inhibition of free radicals.

Furthermore, the obtained results (Table 2) suggest that ethanolic extracts are significantly more effective (z score = 3.41, *p* = 0.001) in inhibition of the lipid peroxidation process when compared to the aqueous extracts. Extract 2E emerged as the most potent (IC_50_ = 220 µg/mL), which is of particular importance when compared to the results obtained for the positive control (butylated hydroxytoluene, (IC_50_ = 13.92 µg/mL). Additionally, the same extract displayed notable effectiveness in the previously presented OH test, which utilizes 2-deoxy-D-ribose as a model of a molecule targeted by free radicals in cells. The highlighted observation allows us to speculate that this extract could have the potential to protect both lipids and carbohydrates in cells from the generated OH• and thus prevent oxidative damage.

To the best of our knowledge, these are the first data describing the potential of hemp (leaf, stem, inflorescence) extracts to reduce/prevent the lipid peroxidation process. A previous study investigated the impact of hemp seed oil on lipid peroxidation in *Drosophila melanogaster* larvae, where it was shown that its application accelerates the formation of MDA and enhances lipid membrane degradation, which was attributed to the elevated concentration of polyunsaturated fatty acids (PUFAs) in hemp oil. Consequently, water and ethanol extracts could represent a more stable alternative, especially in nowadays globally popular hemp-based cosmetics. Specifically, hemp extracts contain no PUFAs, which significantly reduces the risk of contributing to elevated oxidative stress, while, at the same time, as was demonstrated, exhibit significant antioxidant potential [28].

The examined extracts demonstrated a moderate potential to reduce Fe^3+^. The obtained values (Table 2) ranged from 26 to 78 mg ascorbic acid equivalents—AAE/g d.e. Interestingly, the sample 3Aq, which showed modest to moderate antioxidant potential in other assays applied in this study, demonstrated the strongest potential to reduce ferric ions. No statistically significant differences could be observed in the performed FRAP test in relation to the type of extractant used (z score = 0.17, *p* = 0.86). Also, due to the inconsistency in the presentation of the results of the FRAP test, it is difficult to compare these results with the results of other studies that dealt with hemp extracts.

#### 2.2.2. Xanthine Oxidase and Catalase Assay

Xanthine oxidase (XOD), an enzyme that catalyzes the breakdown of purines into uric acid, leads to formation of reactive oxygen species which, in addition to molecular signaling, can often negatively impact health by increasing the levels of oxidative stress. Increased activity of XOD often leads to hyperproduction of uric acid, elevated levels of which can affect the development of a large number of chronic and acute diseases such as gout, cardiovascular system diseases, kidney diseases and metabolic syndromes (diabetes, hyperlipidemia, obesity). Drugs used to treat hyperuricemia are xanthine oxidase inhibitors, such as allopurinol. However, despite its high efficiency, the use of allopurinol often leads to side effects such as allergic reactions, Stevens–Johnson syndrome, hepatitis, renal failure and many others. Therefore, natural alternatives that could exhibit XOD inhibitory activity are increasingly being studied today [29,30,31].

Recent studies have demonstrated the XOD inhibitory effect of cannabidiol, the most abundant cannabinoid in hemp [32]. However, the results of our research show that the polar extracts of hemp leaves also show a potential of XOD inhibition (Table 3).

Specifically, the 5Aq extract showed the most promising activity, inhibiting the XOD activity with IC_50_ = 70 μg/mL, which is of high significance when compared to allopurinol (IC_50_ = 5 μg/mL). Furthermore, the obtained IC_50_ values for the aqueous extracts ranged from 68 to 216 μg/mL, whereas the ethanolic extracts inhibited XOD in the IC_50_ range of 97–129 μg/mL. No statistically significant differences in the XOD inhibition potential between the evaluated types of extracts were noticed (z score = 1.19, *p* = 0.23). Considering these findings, a definitive causative pattern regarding the inhibition of XOD could not be clarified nor could be correlated with prior studies. However, according to the research published so far, polyphenolic compounds (which were also present in our samples) proved to be significant inhibitors of this enzyme [29,33]. Hence, further investigation of these extracts is required, followed by comprehensive chemical characterization. This could facilitate a detailed elucidation of the components within the extracts that exert the most potent inhibition of this enzyme, thus enabling a conclusive determination. Extracts demonstrating XOD inhibition could not only be used in the treatment of hyperuricemia, but could also be part of cosmetic preparations that, by lowering the levels of oxidative stress, positively affect the improvement of skin conditions (especially in some specific diseases, such as psoriasis). It would be necessary to design a suitable formulation of these extracts in order to enable absorption of as many active principles as possible [32].

The antioxidant potential of the extract, calculated via catalase assay, is presented in Table 4.

The aqueous extracts, when tested at higher concentrations, displayed increased antioxidant potency, evidenced by a reduction in catalase levels within the experimental mixtures. Conversely, the ethanol extracts exhibited notably lower antioxidant efficacy.

In a study conducted by Vitorovic et al., 2021 [28], the level of catalase in *Drosophila melanogaster* larvae was monitored under conditions of oxidative stress, after supplementation with hemp seed oil. The levels of this enzyme were reduced with the addition of hemp oil. It was thought that catalase activity was reduced because the oil affected the reduction of peroxide radical levels, which may also be the case with our extracts, as they were shown to be potent scavengers of free radicals in previous assays. This additionally confirms the possibility of using hemp leaf extracts in diseases that are accompanied by high levels of antioxidant stress, as well as in cosmetic preparations that are part of anti-aging collections.

### 2.3. Antihyperglycemic Potential

Diabetes mellitus, a disease representing a global concern, is characterized by the appearance of hyperglycemia and carbohydrate metabolism disorders, which are often accompanied by lipid and protein metabolism disorders. Herbal extracts abundant in phenolic and flavonoid constituents, having the potential to prolong carbohydrate metabolism, are recognized as valuable therapeutic resources. α-amylase and α-glucosidase are pivotal enzymes involved in the initial phase of carbohydrate digestion. Inhibiting these enzymes could potentially attenuate the release of glucose within the intestines, thereby mitigating the organism’s glycemic load [34,35]. To date, research has primarily focused on hemp extracts derived from hemp seeds or hemp protein, while teas have not been explored extensively. Given the ease of preparation and widespread availability of teas, it is imperative to highlight their potential for further investigation.

The results of testing the potential of the hemp extracts to inhibit α-amylase activity (Table 3) indicated a significantly stronger anti-α-amylase potential of aqueous extracts when compared to ethanol extracts (z score = 3.07, *p* = 0.002). The recorded IC_50_ values for aqueous extracts ranged from 87 to 168 μg/mL, whereas the anti-amylase potential of acarbose (positive control) determined under identical experimental conditions was IC_50_ = 4.93 ± 0.33 μg/mL. Although a stronger enzyme inhibitory potential of the evaluated positive control was observed, it should be kept in mind that the pure substance is being compared with a complex mixture (such as herbal extracts) in which there are also compounds without corresponding biological activity. Also, there was a statistically significant correlation of a moderate level between the amount of chlorogenic acid and the anti α-amylase potential (r = −0.55, *p* < 0.05).

A previously conducted study on the influence of different solvents (water, ethanol, hexane and dichloromethane) on the chemical composition of hemp seed extracts found that aqueous extracts were the most potent when it comes to inhibiting α-amylase in vitro, followed by ethanolic extracts, which is in accordance with the results of the current study. Specifically, water extraction led to a higher quantity of polyphenolic constituents, including catechin di-hydrate, cinnamic acid, p-coumaric acid, caffeic acid, benzoic acid and sinapic acid, which are recognized for their α-amylase inhibitory properties [35].

The results of evaluating the anti-α-glucosidase potential of industrial hemp extracts showed a significantly stronger biological activity of ethanolic extracts compared to aqueous extracts (z score = 3.41, *p* = 0.001) (Table 3). The anti-glucosidase potential of the evaluated ethanolic extracts ranged from 59 to 76 μg/mL, which is of high relevance since the anti-glucosidase potential acarbose determined under identical experimental conditions (IC_50_ = 42.87 ± 3.43 μg/mL) directly suggests a strong antihyperglycemic effect of ethanolic extracts of hemp. The obtained results were also consistent with the results of the hemp seed extract research, in which the ethanolic extracts showed the highest in vitro inhibition of α-glucosidase, while the aqueous ones were slightly less potent. Furthermore, the aforementioned study explored the in vivo efficacy of the seed extract, revealing its capacity to reduce postprandial glycemia in rats [35]. A study published by Suttithumsatid et al., 2022 [36] verified the superior anti-α-glucosidase activity of hemp extracts in comparison to isolated CBD and THC. The aforementioned suggests that cannabinoids, although currently gaining the most research attention, might not be the only biologically active class of compounds present in hemp which has the potential to prevent or treat specific conditions. Furthermore, extracts, particularly aqueous formulations, offer a safer alternative due to the minimal presence of potentially toxic cannabinoids [37].

## 3. Materials and Methods

### 3.1. Herbal Material and Extracts Preparation

The herbal material used in the research was represented by commercially available teas based on hemp collected in 2021 (Supplementary Table S1) in the territory of the Republic of Serbia, Slovenia and Austria. Until the preparation of the extracts, the plant material was stored in a dry place at room temperature (25 °C), protected from sunlight. The collected samples contained either hemp leaves or the upper parts of the plant.

Aqueous extracts of the collected samples were prepared in the form of infusions according to the manufacturer’s recommendation. The drug: solvent ratio was 1:10 (*m/m*). The resulting infusions were filtered and evaporated to dryness, after which the extraction yield was quantified (dry extract content—d.e.). Dry extracts were dissolved in distilled water at a concentration of 10% and stored at −20 °C until the experiments were performed. Ethanol extracts were obtained by maceration with 70% (*v*/*v*) ethanol for 24 h (drug: solvent ratio was 1:10 (m/m)) as one of the recognized methods of herbal extract preparation [38]. The resulting macerates were filtered and evaporated to dryness, after which the extraction yield was quantified. The dry extracts were dissolved in 10% (*v*/*v*) dimethyl sulfoxide (DMSO) and stored at −20 °C until the experiment.

### 3.2. Chemical Characterization of Plant Extracts

The preliminary chemical characterization included the quantification of the content of total phenolics and flavonoids in the obtained extracts. The content of total phenolics was determined based on the previously described spectrophotometric method [39]. Phenolics react with the Folin–Ciocalteu reagent to form a blue-colored compound that shows an absorption maximum at 760 nm. The content of total phenolics was expressed as milligrams of gallic acid equivalents per gram of dry extract (mg GAE/g d.e.) based on the previously constructed calibration curve of a gallic acid standard solution [40].

The content of total flavonoids was determined based on the complexation of flavonoids present in the obtained extracts with the AlCl_3_ reagent. The resulting complex shows the maximum absorption at 430 nm [39]. The results were expressed as milligrams of quercetin equivalents per gram of dry extract (mg QE/g d.e.) (based on a previously constructed calibration curve for quercetin) [40].

For the purpose of detailed chemical analysis, the content of caffeic acid (CA), gallic acid (GA), p-coumaric acid (pQA), trans-cinnamic acid (CNA), rosmarinic acid (RA), chlorogenic acid (CHA), ferulic acid (FA), quercetin (Qe), rutin (R) and quercitrin (Qt) was determined using the high performance liquid chromatography (HPLC-DAD, Agilent Technologies 1100, Agilent Technologies, Santa Clara, CA, USA) instrumental technique according to the previously described method [41]. The dried hemp extracts were dissolved in a mixture of equal volumes of used mobile phases, while the separation was carried out using a Nucleosil C18 column (250 mm, i.d. 4.6 mm, 5 µm particle size; Macherey Nagel). As a mobile phase, 1% formic acid (A) and methanol (B) were used, according to a gradient elution program (0 min–10% B; 10 min–25% B; 20 min–45% B; 35 min–70% B; 40 min–100% B; 46 min–10% B), as well as variable flow rate (0–10 min, 1 mL/min; 10–20 min, 0.8 mL/min; 20–30 min, 0.7 mL/min; 30–46 min, 1 mL/min) in order to achieve a more efficient separation. The volume of the injection was 10 μL. Chemical standards of caffeic acid, gallic acid, p-coumaric acid, trans-cinnamic acid, rosmarinic acid, chlorogenic acid, ferulic acid, quercetin, rutin and quercitrin were analyzed under the same experimental conditions in order to obtain calibration curves and other necessary statistical parameters for analyte quantification (Supplementary Table S2). Chromatograms were monitored at three wavelengths: 280 nm for gallic acid, caffeic acid and trans-cinnamic acid; 330 nm for p-coumaric acid, chlorogenic acid, rosmarinic acid, ferulic acid and quercetin and 350 nm for rutin and quercitrin.

### 3.3. Determination of In Vitro Antioxidant Potential

#### 3.3.1. Radical Scavenging Capacity (RSC)

The ability of the prepared extracts to scavenge free radicals was tested according to the previously described spectrophotometric methods using 2,2-dipheny-l-picrylhydrazil (DPPH•), hydroxyl (OH•) and nitroso (NO•) radicals [42,43]. Different concentrations of the evaluated hemp extracts were added to a purple-colored solution of stable DPPH•, and the color change to yellow (which originates from the reduced form—DPPH-H) was monitored spectrophotometrically at 515 nm. The neutralization of OH• radicals, which were generated in the Fenton reaction, was also monitored spectrophotometrically. In this reaction, the degradation of 2-deoxy-D-ribose to malonyl dialdehyde (MDA) occurs, whereby MDA forms a complex with thiobarbituric acid (TBA), the absorbance of which is measured at 532 nm. The ability of the extract to inhibit generated NO• radicals was tested using the Griess reagent, which forms a pink-purple complex with NO•, the absorbance of which is determined at 546 nm.

The radical scavenging capacity (RSC) of extracts of different concentrations was calculated based on the following Equation (1):

RSC (%) = 100 × (1 − Asample/Ablank)(1)

where Asample is the absorbance of the reaction mixture containing tested extracts, and Ablank is the absorbance of the reaction mixture containing no added extracts.

#### 3.3.2. Inhibition of Lipid Peroxidation (LP)

The ability of the hemp extracts to inhibit the process of lipid peroxidation was determined based on a previously published method [42]. A liposomal emulsion (“PRO-LIPO S”) was used as a model of the cell membrane, and the OH• radicals were generated by the Fenton’s reaction. As a product of membrane degradation, MDA is formed, which reacts with thiobarbituric acid, forming a complex with an absorption maximum at 532 nm. All tests were performed in triplicate.

The percentage of inhibition of lipid peroxidation (I%) was calculated based on the following Equation (2):

I (%) = 100 − 100 × (A/A0) (2)

where A0 is the absorbance of the control, and A is the absorbance of the working sample. Based on the calculated I (%) values, the IC_50_ values were determined by application of regression analysis.

#### 3.3.3. Ferric Reduction Antioxidant Potential (FRAP)

The ability of the evaluated extracts to reduce Fe^3+^ to Fe^2+^ was determined according to the method described Lesjak, Beara [44]. Antioxidants at low pH values reduce the iron (III)-2,4,5-tripyridyl-S-triazine complex to the iron (II)-2,4,5-tripyridyl-S-triazine complex, the absorption of which was measured spectrophotometrically at 593 nm. The results were expressed as mg of ascorbic acid equivalents per g of dry extract (mg AAE/g d.e.), based on a previously constructed calibration curve for the antioxidant potential of ascorbic acid measured under the same experimental conditions.

#### 3.3.4. Inhibition of Xanthine Oxidase

The xanthine oxidase (XOD) activity with xanthine as the substrate was measured spectrophotometrically, based on the modified procedure reported by Sweeney, Wyllie [45]. The reaction mixture contained 0.1 M phosphate buffer (pH 7.8), xanthine oxidase from bovine milk (0.25 U/mL) (Sigma-Aldrich X 4500, Steinheim, Germany) and increasing concentrations of the evaluated hemp extracts, allopurinol (positive control) or buffer, which represented sample, positive control or blank, respectively. This mixture was incubated for 10 min at 30 °C, after which xanthin (0.15 mM) (Sigma-Aldrich X 7375) was added. The absorbance rate (dA/min) of this solution that reflects the activity of XOD was then measured kinetically at 295 nm for 3 min. All assays were done in triplicate. The XO inhibitory activity of the plant extracts was expressed as the percentage inhibition of XO in the above assay mixture system based on the following Equation (3):

I (%) = (1 − (dA/min sample − dA/min blank)) × 100(3)

#### 3.3.5. Catalase Assay

Incubation (1 h at 37 °C) of 100 µL of heparinized blood, 300 µL of both H_2_O_2_ and FeSO_4_ was performed in order to induce oxidative stress in whole blood, while diluted sample extracts (sample) at two concentration levels were added to examine their potential to neutralize this process. After incubation, the absorbance rate (dA/min) of this solution (which reflects the activity of CAT) was measured (dA/min) by the kinetic method described by Jelić et al., 2018 [46]. The antioxidant potential (AP) of the examined plant extracts was calculated based on the following Equation (4):

AP (%) = (1 − ((dA/min sample − dA/min blank)/(dA/min os − dA/min blank))) × 100(4)

where blank contained only the physiological solution and heparinized blood.

### 3.4. Determination of In Vitro Antihyperglycemic Potential

#### 3.4.1. Inhibition of α-Amylase

The ability of the hemp extracts to inhibit α-amylase was measured according to the spectrophotometric method described by Kladar, Srđenović [39]. Porcine α-Amylase (final reaction mixture activity 0.6 U/mL), Starch azure^®^ as a substrate (Sigma Aldrich, (Steinheim, Germany)), and sodium phosphate buffer (pH 7.2) with NaCl were used in order to make a reaction mixture. Different concentrations of extracts were added to the mixture, and, after incubation (10 min), the reaction was stopped by acetic acid (50%, m/m). The inhibition percentage was calculated based on the control measurement contained water instead of the tested extract. As a positive control, acarbose was used.

The percentage of enzyme activity inhibition was calculated based on the following Equation (5):

I (%) = 100 − 100 × (Asample − Asample0)/(Acontrol − Acontrol0) (5)

where Asample and Asample0 represent absorbances of reaction mixtures to which extracts were added, with and without enzyme solutions (to remove the influence of colored extracts). Acontrol and Acontrol0 represent absorbances of control solutions with and without enzyme solutions.

#### 3.4.2. Inhibition of α-Glucosidase

The inhibitory activity of hemp extracts on α-glucosidase (from *Saccharomyces cerevisiae*) was tested based on the previously described spectrophotometric method [47]. The reaction mixtures contained glutathione solution (reduced form), phosphate buffer (pH 6.8), α-glucosidase and p-nitrophenyl-α-D-glucopyranoside (pNP-Glu) as a substrate. The final enzyme activity in the reaction mixture was 7.6 U/L. The extracts were added in increasing concentrations to the reaction mixture, whereas acarbose was used as a positive control. The percentage of enzyme inhibition was calculated based on the equation listed in the alpha amylase assay.

### 3.5. Data Processing

The obtained results were processed by Microsoft Office Excel (v2019) and Tibco Statistica (v13.5). Data were analyzed by descriptive statistical methods. The differences between evaluated types of extracts regarding specific quantitative parameters were assessed by the application of the Wilcoxon Matched Pairs Test, in which the level of significance was kept at *p* = 0.05, while the correlation was evaluated by calculation of the Pearson’s correlation coefficient at the same level of statistical significance.

## 4. Conclusions

Hemp leaves, which have long been considered as a waste product, represent a significant resource of polyphenols. Aqueous and ethanolic extracts of hemp teas examined in this research showed a high abundance of phenolic and flavonoid compounds and therefore proved to be excellent scavengers of free radicals. Furthermore, the ethanol extracts were potent inhibitors of the lipid peroxidation process. Also, the evaluated teas were shown to possess antioxidant potential in the catalase test and to be moderate XOD inhibitors. Although the previous would not be sufficient to consider the use of these extracts in therapy instead of XOD inhibitors, the overall antioxidant potential and XOD inhibition makes hemp extracts interesting agents for use in cosmetic preparations with anti-aging effects. It is also necessary to mention that all the results were obtained for teas that were commercially available and that were not uniform regarding herbal material grinding degree, which significantly affected the extraction process.

Aqueous extracts proved to be better in vitro inhibitors of α-amylase, while ethanolic extracts proved to be better in vitro inhibitors of α-glucosidase. All the conducted tests indicate the great potential of these extracts for the treatment of various diseases and conditions, especially those caused by oxidative stress (insulin resistance, diabetes, obesity, cardiovascular diseases). Also, hemp extracts obtained with polar solvents (especially water) represent a safer product for consumption, due to their minimal affinity to extract liposoluble cannabinoids. Furthermore, it could be of particular interest to study different fractions of obtained extracts by bioactivity-guided fractionation in order to define compounds, or classes of compounds, responsible for specific effects and make their utilization more efficient.

## Figures and Tables

**Table 1:** The results of preliminary (total phenolics and flavonoids) and detailed (phenolic acids, flavonoids and flavonoid glycosides) chemical characterization of hemp extracts.

Sample	Extraction Yield (%)	Preliminary Chemical Characterization		Detailed Chemical Characterization (μg/mg of d.e.)									
		TPC * (mg GAE/g d.e.)	TFC ** (mg QE/g d.e.)	GA	CA	CNA ^a^	pQA ^b^	Qe	CHA	RA	FA	R	Qt
1E	15.73	57.44 ± 2.34	9.48 ± 0.23	<LOD ***	0.052 ± 0.002	<LOD	0.009 ± 0.001	<LOD	2.661 ± 0.133	<LOD	<LOD	<LOD	0.210 ± 0.010
2E	22.18	58.35 ± 4.1	33.26 ± 0.51	<LOD	0.104 ± 0.005	<LOD	<LOD	<LOD	<LOD	<LOD	<LOD	<LOD	<LOD
3E	9.44	35.02 ± 3.5	6.4 ± 0.28	<LOD	0.052 ± 0.002	<LOD	<LOD	<LOD	<LOD	<LOD	0.190 ± 0.011	<LOD	<LOD
4E	19.99	62.57 ± 4.41	16.88 ± 0.51	<LOD	0.559 ± 0.028	<LOD	<LOD	<LOD	<LOD	<LOD	<LOD	<LOD	<LOD
5E	13.01	47.1 ± 4.41	9.48 ± 0.45	<LOD	0.166 ± 0.081	0.081 ± 0.009	0.149 ± 0.015	<LOD	<LOD	<LOD	<LOD	<LOD	<LOD
1Aq	28.73	38.64 ± 2.67	9.88 ± 0.23	<LOD	0.166 ± 0.008	<LOD	<LOD	<LOD	0.068 ± 0.003	<LOD	<LOD	<LOD	<LOD
2Aq	23.23	49.72 ± 2.98	17.88 ± 0.45	<LOD	0.115 ± 0.006	<LOD	<LOD	<LOD	<LOD	<LOD	<LOD	<LOD	<LOD
3Aq	12.7	24.81 ± 2.14	7.56 ± 0.34	<LOD	0.102 ± 0.005	0.102 ± 0.011	<LOD	<LOD	0.119 ± 0.006	<LOD	<LOD	<LOD	<LOD
4Aq	29.56	71.74 ± 7.25	13 ± 0.45	<LOD	0.054 ± 0.003	0.052 ± 0.006	<LOD	<LOD	<LOD	<LOD	<LOD	<LOD	<LOD
5Aq	17.48	50.19 ± 4.56	17.12 ± 1.01	<LOD	<LOD	<LOD	<LOD	<LOD	0.048 ± 0.002	<LOD	<LOD	<LOD	<LOD

* Total phenolic content. ** Total flavonoid content. *** LOD—limit of detection. ^a^ trans-cinnamic acid. ^b^ p-coumaric acid.

**Table 2:** In vitro antioxidant potential (free radicals neutralization potential, ferric reduction potential and inhibition of lipid peroxidation).

Sample	DPPH (IC_50_-µg/mL)	OH (IC_50_-µg/mL)	NO (IC_50_-µg/mL)	FRAP (mg AAE */g.d.e)	LP (IC_50_-µg/mL)
1Aq	158.76 ± 6.35	733.42 ± 36.67	1002.23 ± 65.14	31.20 ± 6.87	ND
2Aq	385.86 ± 30.87	ND **	954.75 ± 62.06	25.68 ± 10.93	676.58 ± 33.35
3Aq	80.46 ± 4.83	481.02 ± 30.25	359.26 ± 23.35	77.35 ± 11.24	ND
4Aq	49.71 ± 1.49	256.15 ± 25.68	457.14 ± 29.71	55.73 ± 9.14	ND
5Aq	76.41 ± 6.54	450.36 ± 22.98	459.69 ± 29.88	32.63 ± 7.15	625.21 ± 30.14
1E	38.57 ± 2.81	271.49 ± 14.53	1126.25 ± 73.21	44.96 ± 3.98	557.36 ± 28.74
2E	35.02 ± 2.03	222.22 ± 10.25	ND	29.36 ± 3.55	220.21 ± 11.12
3E	49.71 ± 3.41	317.07 ± 15.89	998.65 ± 64.91	58.43 ± 8.23	633.93 ± 30.21
4E	49.8 ± 3.21	300.24 ± 13.58	1100.36 ± 71.52	49.38 ± 7.76	886.42 ± 41.21
5E	35.33 ± 1.89	224.56 ± 10.24	1263.25 ± 82.11	50.93 ± 8.53	219.56 ± 10.89

* AAE—ascorbic acid equivalents. ** ND—not detected.

**Table 3:** The ability of hemp extracts to inhibit biologically important enzymes; antihyperglycemic and antiuric potential.

Sample	α-Amylase (IC_50_ µg/mL)	α-Glucosidase (IC_50_ µg/mL)	XOD (IC_50_, µg/mL)
1E	319.47 ± 15.97	66.87 ± 4.35	114.73 ± 9.24
2E	154.21 ± 7.71	60.86 ± 3.96	97.09 ± 7.77
3E	150.28 ± 7.51	66.21 ± 4.30	104.82 ± 8.70
4E	251.09 ± 12.55	75.82 ± 4.93	129.17 ± 9.64
5E	210.62 ± 10.53	58.76 ± 3.82	100.12 ± 7.81
1Aq	154.44 ± 7.72	90.3 ± 5.87	215.15 ± 17.21
2Aq	164.76 ± 8.24	ND *	173.25 ± 14.21
3Aq	86.77 ± 4.34	152.18 ± 9.89	116.09 ± 8.82
4Aq	126.91 ± 6.34	230.43 ± 14.98	113.89 ± 9.57
5Aq	167.75 ± 8.39	69.6 ± 4.52	67.92 ± 5.16

* ND—not detected.

**Table 4:** Antioxidant potential (%) of hemp extracts at two concentration levels in the catalase assay.

Sample	Concentrations of Extracts			
	35 µg/mL E *	85 µg/mL E	35 µg/mL Aq **	85 µg/mL Aq
1	27.44 ± 1.37	37.22 ± 1.86	67.24 ± 3.36	68.10 ± 3.40
2	17.84 ± 0.89	0.86 ± 0.04	17.84 ± 0.89	68.95 ± 3.45
3	ND ***	4.46 ± 0.22	22.47 ± 1.12	59.86 ± 2.99
4	29.85 ± 1.49	26.07 ± 1.30	29.85 ± 1.49	36.54 ± 1.83
5	7.38 ± 0.37	8.75 ± 0.44	31.39 ± 1.57	42.02 ± 2.10

* E—ethanol extracts. ** Aq—aqueous extracts. *** ND—not detected.

## Data Availability

The data are contained within the article.

## Supplementary Materials

The following supporting information can be downloaded at: https://www.mdpi.com/article/10.3390/plants13121630/s1.
