# Supplementary material for: The Potential of Hemp Extracts to Modify the Course of Oxidative-Stress Related Conditions
Source: Plants (Basel). 2024 Jun 13;13(12):1630. doi: 10.3390/plants13121630 (PMC11207586; doi:10.3390/plants13121630)
Supplement: Supplementary file 1 [file plants-13-01630-s001.zip › supplementary Table S1 Sample details.pdf]

Supplemetary Table S1. Sample details

Sample	Name in the original language	Name in english	Type	Producer	Purchased in
1	Čaj od industrijske konoplje	Hemp tea	dried leaves	Svet Konoplje	Serbia
2	Hanf blatter	Hemp leaves	dried leaves	SonnentoR	Austria
3	Konopljin čaj	Hemp tea	dried upper parts	BEHEMPY	Slovenia
4	Konopljin čaj	Hemp tea	dried upper parts	Taste	Slovenia
5	Bio konopljin čaj	Bio hemp tea	dried hemp leaves	BioMlin Strašar d.o.o.	Slovenia
