# Supplementary material for: The Potential of Hemp Extracts to Modify the Course of Oxidative-Stress Related Conditions
Source: Plants (Basel). 2024 Jun 13;13(12):1630. doi: 10.3390/plants13121630 (PMC11207586; doi:10.3390/plants13121630)
Supplement: Supplementary file 1 [file plants-13-01630-s001.zip › supplementary Table S2 Validation parameters.pdf]

Supplementary Table S2. Validation parameters

Analyte	Equation	R^2	LOD (µg/g of dry extract)	LOQ (µg/g of dry extract)	U (%)
trans-cinnamic acid	y=7.9398x+6.5653	0.999	1.5	5	11
caffeic acid	y=6.4807x+1.4903	0.999	1.5	10	5
p-coumaric acid	y=5.5894x+12.473	0.999	1.5	10	10
quercetin	y=9.4489x+8.3990	0.998	1.5	10	7
chlorogenic acid	y=2.9003x+4.6876	0.999	6.5	15	5
rosmarinic acid	y=3.5125x+0.3322	0.998	8.0	25	6
ferulic acid	y=1.6072x-5.5118	0.998	4.5	12	6
gallic acid	y=3.0497x+5.4494	0.999	2.5	15	15
rutin	y=2.1224x+5.4787	0.999	8.0	25	8
quercitrin	y=2.4017x-2.8372	0.999	6.5	25	5

R^2 Coefficient of determination

LOD Limit of detecton

LOQ Limit of quntification

U Expanded measurment uncertainty (k=2)
